# Engineering Approaches for the Development of Antimicrobial Peptide-Based Antibiotics

**DOI:** 10.3390/antibiotics11101338

**Published:** 2022-09-30

**Authors:** Su-Jin Kang, So Hee Nam, Bong-Jin Lee

**Affiliations:** 1 College of Pharmacy, Dongduk Women’s University, Seoul 02748, Korea; 2 Research Institute of Pharmaceutical Sciences, College of Pharmacy, Seoul National University, Seoul 08826, Korea

**Keywords:** antimicrobial peptides (AMPs), multidrug-resistant (MDR) bacteria, antibiotics, engineering approaches

## Abstract

Antimicrobial peptides (AMPs) have received increasing attention as potential alternatives for future antibiotics because of the rise of multidrug-resistant (MDR) bacteria. AMPs are small cationic peptides with broad-spectrum antibiotic activities and different action mechanisms to those of traditional antibiotics. Despite the desirable advantages of developing peptide-based antimicrobial agents, the clinical applications of AMPs are still limited because of their enzymatic degradation, toxicity, and selectivity. In this review, structural modifications, such as amino acid substitution, stapling, cyclization of peptides, and hybrid AMPs with conventional antibiotics or other peptides, will be presented. Additionally, nanodelivery systems using metals or lipids to deliver AMPs will be discussed based on the structural properties and action mechanisms of AMPs.

## 1. Introduction

Antibiotics have been considered a miracle drug against various bacterial infections in both humans and animals for more than 100 years. The first antibiotic, salvarsan, in 1910 and the discovery of penicillin in 1928 led to the antibiotic era of the 20th century [1]. However, the easy access to and overuse of antibiotics not only in the hospital, but also in the farming or livestock industries, have driven and accelerated the development of resistance against traditional antibiotics in bacteria. Moreover, the emergence and worldwide spread of multidrug-resistant (MDR) bacteria, such as methicillin-resistant *Staphylococcus aureus* (MRSA), vancomycin-resistant *Enterococci* (VRE), and carbapenem-resistant *Enterobacteriaceae* (CRE), are a major concern in global health care [2,3,4,5]. Effective therapeutic strategies are no longer relevant, and an urgent challenge is the development of novel and potent antibacterial agents. Antimicrobial peptides (AMPs) have captured attention as alternative solutions to combat diverse infections caused by drug-resistant bacteria [6].

AMPs are small polypeptide molecules that are produced by all living organisms to protect a host from pathogenic microbes. AMPs play a crucial role in defending against bacterial, viral, and fungal infections, as well as in adaptive immunity. These peptides display remarkable structural and functional diversity and have action mechanisms different to those of existing antibiotics [7]. These characteristics make AMPs exhibit potential capacity as prospective therapeutics to replace conventional antibiotics and a new treatment for MDR bacterial infections.

Based on the AMP database (http://aps.unmc.edu/AP/), more than 3000 AMPs from six life kingdoms have been described to date. However, there are very few clinical applications of AMPs as antibiotics thus far, and only 96 clinical studies for AMPs can be found in the clinical trial database (https://www.clinicaltrails.gov). In addition, their applications as drugs are limited to skin infections (Table 1) [4,8]. Peptide drugs face several obstacles in bringing new AMP therapeutics to the market [2,9,10]. AMPs exhibit undesirable characteristics, such as proteolytic digestion, toxicity to eukaryotic cells, and inefficient delivery to the target site. To introduce novel AMP-based drugs to clinics, chemical and/or physical engineering, such as size reduction, control of hydrophobicity, specific amino acid substitution, de novo design, and prodrugs have been suggested. This review focuses on engineering approaches, including structural modifications, conjugation systems, and nanodelivery systems, in the development of new antimicrobial peptide-based antibiotics.

## 2. Structural Characteristics, Classification, and Action Mechanisms of AMPs

AMPs are relatively short peptides that have fewer than 60 amino acid residues. The number of amino acid residues in AMPs is typically between 12 and 40. Their surfaces show a positive charge in the range of +2 to +9 because basic amino acids, such as arginine, lysine, and histidine, are usually abundant. These cationic peptides selectively interact with the negatively charged membranes of bacteria, while they interact weakly with the relatively neutral eukaryotic membrane. In addition, many of them have 40% to 60% hydrophobic residues, which are crucial for penetrating the hydrophobic membrane of bacteria. However, several anionic AMPs exist as well, in which acidic amino acids, such as aspartic acid and glutamic acid, are abundant. Most AMPs are usually unstructured in aqueous solutions, but their interaction with the bacterial membrane causes AMPs to form amphipathic structures by putting hydrophobic residues on one side of the peptide and hydrophilic residues on the other side [7,11,12,13,14].

AMPs can be generally classified into four groups based on their secondary structures: (i) α-helical peptides, (ii) β-sheet peptides, (iii) extended peptides, and (iv) loop peptides [10,15,16,17]. α-helical peptides usually have a linear structure without cysteine. They adopt a random coil structure in aqueous solutions, but change their conformation in a hydrophobic membrane environment by forming amphipathic helices, finally leading to the disruption of the bacterial membrane. Peptides in this group are the most investigated and are a representative class of AMPs, including magainin, cecropin, and pexiganan [18]. The β-sheet peptides form more ordered and more rigid structures because they have intramolecular disulfide bridges between an antiparallel β-sheet. Drosocin and histatin 5 are included in this group [19,20]. The third group of AMPs, extended peptides, are relatively unstructured, rare, and less studied. They have specific amino acids, such as proline, tryptophan, arginine, and histidine. Indolicidin is a tryptophan/proline-rich extended peptide, and Bac5 and Bac7 are proline/arginine-rich extended peptides [21]. Finally, loop peptides, such as microcin and bactenecin, form a loop structure with one disulfide bond [18].

**Figure 1:**
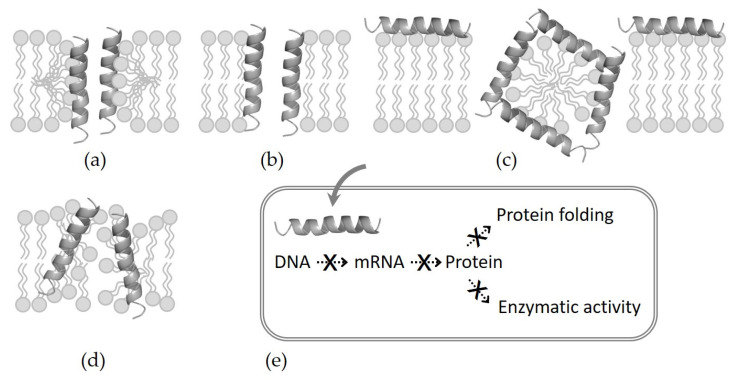
Action mechanisms of AMPs. (**a**) Barrel–stave model, (**b**) toroidal pore model, (**c**) carpet model, (**d**) aggregate model, and (**e**) inhibition of vital cell processes after passing through the bacterial membrane.

The action mechanisms of AMPs are complex and still controversial, but the most accepted mechanism can be explained by the model of how the interaction of peptides with the bacterial membrane leads to the disruption of the membrane’s integrity. Selective binding is generally related to structural properties, such as size, charge, hydrophobicity, secondary structure, and amphiphilic characteristics. Four models have been widely proposed for killing bacteria through membrane permeabilization (Figure 1) [2,22,23]. (i) Barrel–stave model: The amphipathic α-helical peptides aggregate and form barrel-like bundles in the bacterial membranes. Gradually, the channel size is expanded, resulting in the outflow of the intracellular material and subsequent cell death. Almethicin isolated from *Trochoderma rivide* fungus is the most well-known peptide with a barrel–stave mechanism [24]. (ii) Toroidal pore model: AMPs insert themselves vertically into the bacterial membrane, inducing a lipid monolayer to be distorted and resulting in the formation of a toroidal pore. Representative examples of this model include arenicin, lacticin Q, and magainin [23]. (iii) Carpet model: AMPs accumulate and align in parallel with the surface of the bacterial membrane, forming a “carpet”. By forming micelles and pores, they act as detergents to collapse the bacterial membrane. LL-37, a cathelicidin-related peptide, in humans and the amphipathic dermaseptin peptide produced in phyllomedusine frog skin act using this mechanism [23,25]. (iv) Aggregate model: AMPs, such as indolicidin, are embedded inward in the bacterial membrane to form aggregates of peptides and membrane lipids, leading peptides to move across the membrane. After entering the cell, AMPs exert various nonmembrane and nondirect effects synergistically with membrane disruption. They impede the synthesis of DNA, mRNA, and proteins, and inhibit the synthesis of the cell wall and the activation of immune cells or enzymes. As a result, AMPs also show antibacterial, antifungal, anticancer, and immunomodulatory activity [2,9,26].

## 3. Structural Modification

### 3.1. L- to D-Amino Acid Substitution

**Figure 2:**
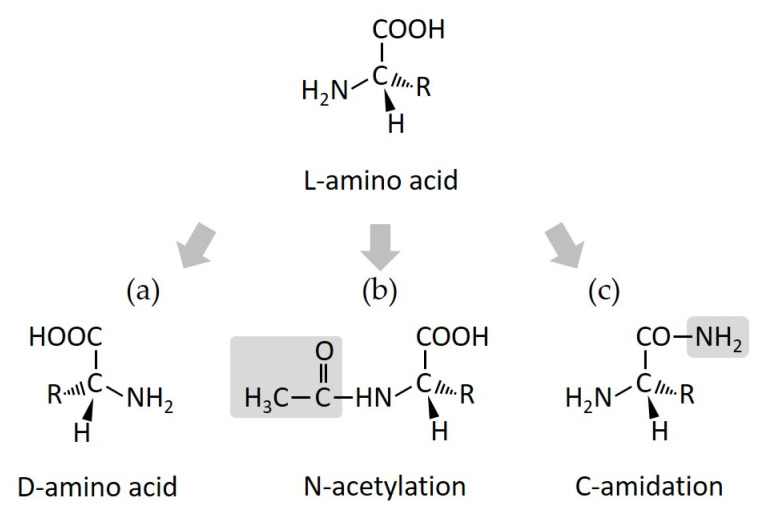
Structural modification. (**a**) L- to D-amino acid substitution, (**b**) N-acetylation, and (**c**) C-amidation.

D-amino acid substitution in order to replace natural L-amino acids in AMPs is a commonly used strategy for improving peptide stability against protease digestion (Figure 2), because human and microbe proteases exclusively recognize L-amino acids, rather than D-amino acids [3,27,28,29,30]. The D-amino acid-substituted derivative from the polybia-CP peptide designed by Jia et al., showed improved stability by approximately six times against trypsin and chymotrypsin [31]. In addition, this method can increase the retention time of antimicrobial activity and sometimes promote the minimal inhibitory concentrations (MICs) of AMPs [32,33,34,35]. Leu et al., synthesized peptides, derived from cationic AMP Pep05, by substituting L-amino acid residues with D- and unnatural amino acids, which resulted in increased activities and decreased toxicities. Among them, the UP09 peptide exhibited improved stability against trypsin. Fifteen percent of the UP09 peptide remained 18 h after digestion, while the original peptide was degraded in one hour after digestion [36].

### 3.2. Terminal Acetylation and Amidation

Usually, the N-terminal acetylation of AMPs increases the helicity of peptides and prevents enzymatic degradation, and its C-terminal amidation enhances structural stability and antimicrobial activity (Figure 2) [37,38,39,40]. In a report by Alvares et al., the L1A peptide adopts a more helical conformation when its N-terminus is acetylated [37], and Li et al. designed an L163 analog by amino-terminal acetylation, which exhibited higher stability against trypsin degradation [41]. The C-terminal amidation of the Mac1 peptide also plays an important role in maintaining a stable α-helical structure in contact with micelles and results in higher antibacterial activity [42]. Upon amidation, esculentin-2 peptide analogs showed increased antimicrobial activity and selectivity [43]. Moreover, AMPs can be modified in the N-terminus and C-terminus at the same time. Tachyplesin I, a peptide with C- and N-terminal modifications, was resistant to proteolytic degradation in human serum and exhibited a more potent cytotoxic effect on cancer cells and better pharmacokinetic properties [44].

### 3.3. Stapled Peptides

**Figure 3:**
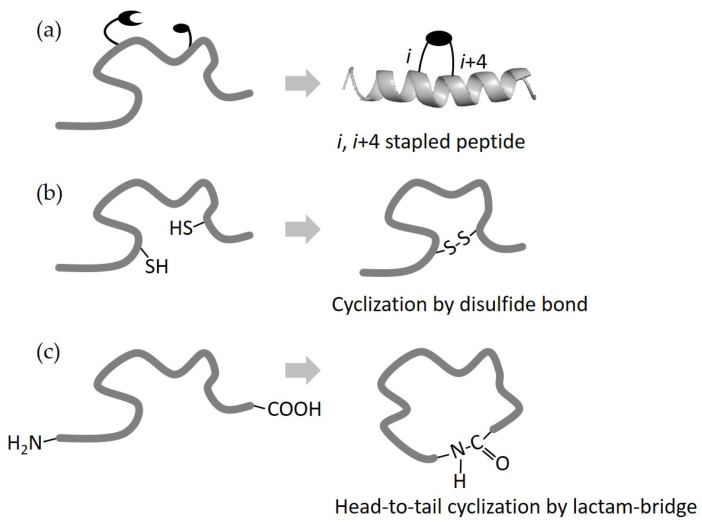
Structural modification. (**a**) Stapled peptide with an external brace across one turn (*i*, *i* + 4), (**b**) cyclization by a disulfide bond, and (**c**) cyclization by a lactam bridge.

Stapling is another technique for improving the antimicrobial activity and stability of AMPs by helix stabilization (Figure 3). Stapled peptides are forced to form an α-helical structure in which the side chains are cross-linked by methods such as C–H activation, tryptophan condensation, and ring-closing metathesis [45]. This rigid helical conformation increases the activity of AMPs and their resistance to proteases by hiding proteolytic targets [45,46,47]. Hirano et al., designed and synthesized magainin 2 derivatives with stapled hydrocarbon side chains, which showed higher antimicrobial activity without exerting significant hemolytic activity [48]. The Hu group synthesized hydrocarbon side-chain-stapled analogs of the ascaphin 8 peptide, which exhibited improved stability and biological activities [49].

### 3.4. Peptide Cyclization

Peptide cyclization is a particularly promising approach for improving both the stability and bioactivity of AMPs. Similar to side-chain stapling with one or more external braces, peptide cyclization also contains cross-linking constructions with disulfide bonds and those with lactam bridges (Figure 3) [50,51]. A study by Neubauer et al., suggested that a disulfide-cyclized ultrashort cationic lipoprotein reduced cytotoxicity and exhibited improved selectivity between *Candida* sp., Gram-positive strains, and normal cells [52]. By side-chain lactam cyclization, Scala et al. increased the stability of peptides derived from the bovine lactoferrin C-lobe [50].

As well as the methods mentioned above, various structural modifications based on the characteristics, structures, and action mechanisms of AMPs have been investigated. To modulate the hydrophobicity or charge of AMPs, some positions can be substituted with other specific amino acids, not only with D-amino acids, as discussed above [2]. For cost reduction in clinical applications, the size of AMPs could be shortened. Furthermore, de novo-designed synthetic AMPs are considered as a potential class of antibiotics [53,54].

## 4. Conjugation System

### 4.1. Hybrid Peptide (Peptide-Peptide Conjugate)

**Figure 4:**
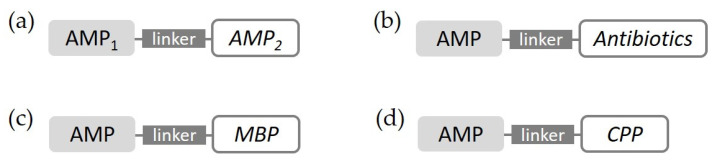
Conjugation system. (**a**) Hybrid peptide, (**b**) antibiotic–peptide conjugate, (**c**) AMPs conjugated with membrane-binding peptides (MBP), and (**d**) AMPs conjugated with cell-penetrating peptides (CPPs).

Peptide-peptide conjugates, in which two or more different antimicrobial peptides are merged into one, have been reported to produce stronger activity, even against drug-resistant bacteria. In this case, important points can be taken from the structure or sequence of well-known peptides for biological action, and they are combined via a linker (Figure 4) [55,56]. A hybrid peptide derived from BMAP-27 and OP-145 as two parent α-helical peptides exhibited a broad spectrum of antimicrobial activity, even against MDR bacterial strains, and reduced toxicity toward eukaryotic cells [57]. The triple hybrid from cecropin A, LL-37, and magainin II, which all have been well-studied antimicrobial peptides for a long time, showed greater antimicrobial activities than those of the parent AMPs [58].

### 4.2. Antibiotic-Peptide Conjugate

Antibiotic-peptide conjugates (APCs), a hybrid of existing antibiotics and AMPs, can also be good candidates in conjugation systems with AMPs (Figure 4). The synergistic antimicrobial activities of APCs have been studied to overcome the well-known shortcomings of conventional antibiotics or antimicrobial peptides [59]. For bacteria-targeting therapy, UBI_29–41_ was attached to chloramphenicol (CAP), a well-known antibiotic. In vitro studies demonstrated the enhanced antibacterial effects of CAP-UBI_29–41_ selectively on *S. aureus* and *E. coli*, showing reduced toxicity to normal cells [60]. A novel hybrid peptide, V-IDR1018, a conjugate of vancomycin and an innate defense regulator peptide, exhibited potent activity and showed no susceptibility to antimicrobial resistance. Vancomycin–magainin conjugates designed by Breukink et al. showed an increase in antimicrobial activity against VRE when compared with vancomycin alone [61]. Similarly, vancomycin is often used for antibiotic–peptide conjugates with FDA approval, such as Telvancin and Dalbavancin, as shown in Table 1 [62].

### 4.3. AMP-Particular Peptide Conjugate

AMPs can be conjugated with particular peptides, such as membrane-binding peptides or cell-penetrating peptides (Figure 4) [3]. Combined with these functionalized peptides, AMPs can adopt a special function and cover up their weaknesses. Two kinds of smart chimeric peptides (SPCs), which connected LPS-binding peptide (LBP) 14 with marine AMP-N6, displayed more potent antibacterial activity against MDR *Escherichia coli* and more effectively neutralized lipopolysaccharide toxicity than the peptide alone, both in vitro and in vivo [63]. AMPs, such as magainin and M15, conjugated with cell-penetrating peptide (CPP) showed a 4- to 16-fold increase in antimicrobial activity against G-negative bacteria by enhanced membrane permeabilization and translocation [64]. Two conjugates of CPP and N2 peptide, which are active against *Salmonella typhimorium*, such as B6N2 and T11N2, showed lower MICs at acidic pH and higher killing rates than N2 alone and other antibiotics, such as ciprofloxacin and ceftriaxone [65].

Except for the conjugates with known antibiotics or particular peptides, AMPs can be conjugated with other active molecules, such as fatty acids, anticancer drugs, photosensitizers, antibodies, and so on. Fatty acid conjugated peptides can exhibit enhanced antibacterial activity and reduced eukaryotic cytotoxicity by promoting interaction with bacterial cell membranes [66]. Conjugation with a photosensitizer helps AMPs to effectively kill resistant bacteria strains because the photosensitizer produces reactive oxygen species (ROS) after exposure to a particular light. Antibody—AMP conjugates promote selectivity and specificity to the target [67]. Thus, conjugates of AMPs not only improve the characteristics of AMPs themselves, but also expand the activity spectrum of AMPs.

## 5. Nanodelivery System

**Figure 5:**
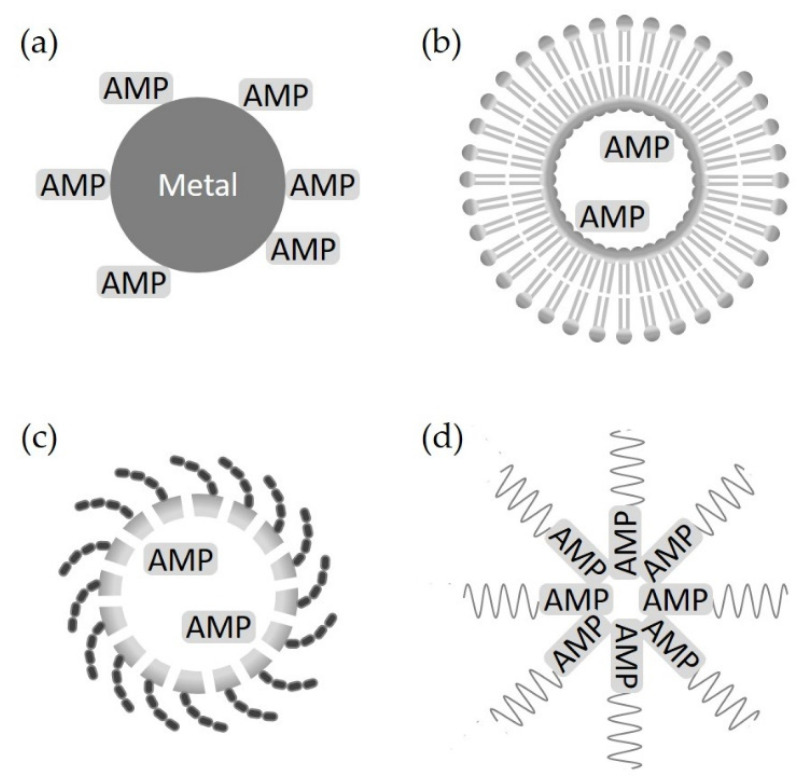
Nanodelivery system. (**a**) Metal nanoparticles conjugated with AMPs, (**b**) lipid-based nanoparticles conjugated with AMPs, (**c**) polymeric-based nanostructure with encapsulated AMPs, and (**d**) self-assembled AMPs.

AMP engineering using nanotechnology provides an effective solution for the major problems of AMPs, such as instability, toxicity, and target selectivity [4,68,69]. Nanotechnology in drug development from AMPs means the conjugation of AMPs and nanoparticles, a type of carrier to deliver AMPs (Figure 5).

### 5.1. Metal Nanoparticles

Nanoparticles containing metal, such as silver or gold, can be conjugated to AMPs through the processes of physisorption or chemisorption [70]. First, silver nanoparticles (AgNPs) themselves have antibacterial activities against G-negative and G-positive bacteria, including multidrug-resistant microorganisms. Thus, a synergistic effect in the combined use of AgNPs and AMPs can be expected [5,71,72]. AgNPs conjugated with AMPs, such as protegrin-1, indolicidin, protamine, and histones, enhanced their antimicrobial potential and effectively reduced the toxicity of membranolytic AMPs [73]. It has been reported that a conjugate of andersonin-Y1 peptide and AgNPs exhibited a nearly 10-fold increase in antibacterial activity against multidrug-resistant strains [74]. Similarly, gold nanoparticles (AuNPs) can also be conjugated with AMPs to overcome the instability of peptides and their low penetrability into host cells. A AuNP-Apt–HPA3P^His^ conjugate, in which the HPA3P^His^ peptide was loaded onto a gold nanoparticle-DNA aptamer, was designed by Lee et al., and this conjugate improved the permeability of HPA3P^His^ and eliminated bacteria a few hours after treatment without toxicity to the host [75]. Another study by Casciaro et al. showed that a new AuNP–esculentin(1-21) conjugate demonstrated increased activity by ~15-fold against *Pseudomonas aeruginosa* without toxicity to human keratinocytes and was significantly more resistant to proteolytic digestion [76].

### 5.2. Lipid-Based Nanoparticles

Lipid-based nanoparticles (LNPs) have served as delivery systems for AMPs due to their several favorable characteristics, such as physical and chemical stability, biocompatibility, and low cytotoxicity to normal cells [5,69,77]. Liposomes are the most well-known and most widely applied nanocarriers for drug delivery and clinical applications. A study by Cantor et al. proved that the antibacterial activity of a peptide encapsulated into nanoliposomes was increased by approximately 2000-fold against *Listeria monocytogenes* [78]. Nanosized liposomal formulations of LL-37 and indolicidin showed less toxicity and improved activity [79]. In addition to liposomes, LNP systems for AMPs include micelles, dendrimers, polymeric nanoparticles, and microspheres [69].

### 5.3. Polymer-Based Nanostructures

Polymers are macromolecules composed of many repeated specific units and are used widely in medicinal applications due to their easily modified and flexible physicochemical properties. Polymeric nanoparticles are one of the popular formulations in the size range of 50 to 100 nm, such as chitosan, dextran, polyethylene glycol (PEG), and poly(lactide-co-glycolic acid) (PLGA). Polymeric nanostructures containing AMPs have advantages to increase their stability and consequently improve their antimicrobial performance [80]. Almaaytah et al., encapsulated a potent ultrashort AMP named RBRBR in chitosan-based nanoparticles (CS-NPs), resulting in potent antimicrobial effects against MDR and biofilm-forming bacteria with negligible systemic toxicity and reduced synthetic costs [81]. A PEG hydrogel coating with covalently attached HHC10, which is an AMP acting against MDR pathogens, stabilized the peptide against proteolytic degradation and increased its bactericidal activity [82].

### 5.4. Self-Assembling AMPs

Self-assembled peptide nanomaterials, which are induced self-assemblies of AMPs into nanoparticles, are emerging as an effective approach for the improvement of AMP stability and resistance to degradation [3,83]. By forming liposome-like assemblies, they form a stable structure and protect themselves against proteases, resulting in increased activity. Malini et al., demonstrated the enhanced antimicrobial activity of the self-assembled LL-37 peptide with the amphiphilic lipid glycerol monooleate [84]. The C-terminally myristoylated HD5-assembled nanobiotic displayed significantly improved broad-spectrum antibacterial activity in vitro and selective toxicity against *E. coli* and MRSA, with negligible hemolytic activity and low toxicity [85]. In addition, self-assembling AMPs could play roles as vaccine adjuvants to boost immunogenicity or as delivery carriers for antigenic proteins.

The materials and applications of nanotechnology are uncountable, not only in the development of AMP-based antibiotics, but also in clinical therapeutics. Inorganic materials, such as carbon nanotubes and magnetic nanoparticles, and organic materials, such as cyclodextrin and tetrahedral framework nucleic acid, can be adopted for improving the characteristics of AMPs and gaining the effect of targeting and controlled drug release. Recently, nano-fibers and nano-tapes have been effectively applied to AMPs as well [80,86].

## 6. Conclusions

The inappropriate and excessive use of antibiotics has resulted in severe problems caused by drug-resistant bacteria, particularly in developing countries [2,3]. The need for the development of alternative therapeutics has increased throughout the last three years of the COVID-19 pandemic. AMPs have been considered as promising new antibacterial agents that can replace conventional antibiotics because they show broad-spectrum antimicrobial activities and low probability to develop resistance [2,12,14]. However, very few antimicrobial peptide-based antibiotics are used in the narrow areas of clinical applications because of problems such as proteolytic degradation, selectivity, toxicity to mammalian cells, size, and high cost. In order to overcome these obstacles and enhance their antibacterial activity for the development of AMPs as antibiotics, various attempts have been made chemically and/or physically. Among them, engineering approaches are considered effective strategies. Structural engineering, including L- to D-amino acid substitution, terminal acetylation and amidation, and cyclization, prevents protease digestion and enhances bioactivity [31,36]. By stapling peptides, AMPs strengthen their helicity, thus improving their activities [45,46,47]. Several conjugates, such as hybrid peptides and APCs, take advantage of peptides and existing antibiotics, which act synergistically [55,57]. For stability, toxicity, and target selectivity, nanotechnologies that apply a metal or liposome to AMPs provide intelligent solutions [4,5,68,69]. Many studies using this kind of engineering have proven its effectiveness against MDR pathogens. It is expected that new therapeutics based on AMPs by engineering approaches will substitute resistance-acquired antibiotics in the near future.

## Figures and Tables

**Table 1:** Some antimicrobial peptide drugs approved by the FDA.

Name	Trade Name	FDA Approval	Type	Administration	Application	Antimicrobial Activity
Vancomycin	Vanocin	1983	Heptapeptide	Oral	Bacterialinfections	G-positive bacteria
Bacitracin	Baciim	1997	Cyclic peptide	Topical	Skin and eye infections	G-positive bacteria
Daptomycin	Cubicin	2003	Cyclic lipopeptide	Intravenous	Skin infections	G-positive bacteria
Telavancin	Vibativ	2009	Lipoglycopeptide	Intravenous	Skin infections	G-positive bacteria
Oritavancin	Orbactiv	2014	Lipoglycopeptide	Intravenous	Skin infections	G-positive bacteria
Dalbavancin	Dalvance	2014	Lipoglycopeptide	Intravenous	Skin infections	G-positive bacteria

## Data Availability

Not applicable.

## References

[1] Hutchings M.I., Truman A.W., Wilkinson B. (2019). Antibiotics: Past, present and future. Curr. Opin. Microbiol..

[2] Rima M., Rima M., Fajloun Z., Sabatier J.M., Bechinger B., Naas T. (2021). Antimicrobial Peptides: A Potent Alternative to Antibiotics. Antibiotics.

[3] Zhang C., Yang M. (2022). Antimicrobial Peptides: From Design to Clinical Application. Antibiotics.

[4] Carratalá J.V., Serna N., Villaverde A., Vázquez E., Ferrer-Miralles N. (2020). Nanostructured antimicrobial peptides: The last push towards clinics. Biotechnol. Adv..

[5] Makowski M., Silva Í.C., Pais do Amaral C., Gonçalves S., Santos N.C. (2019). Advances in Lipid and Metal Nanoparticles for Antimicrobial Peptide Delivery. Pharmaceutics.

[6] Liu Y., Shi J., Tong Z., Jia Y., Yang B., Wang Z. (2021). The revitalization of antimicrobial peptides in the resistance era. Pharmacol. Res..

[7] Van ‘t Hof W., Veerman E.C., Helmerhorst E.J., Amerongen A.V. (2001). Antimicrobial peptides: Properties and applicability. Biol. Chem..

[8] Arsene M.M.J., Jorelle A.B.J., Sarra S., Viktorovna P.I., Davares A.K.L., Ingrid N.K.C., Steve A.A.F., Andreevna S.L., Vyacheslavovna Y.N., Carime B.Z. (2022). Short review on the potential alternatives to antibiotics in the era of antibiotic resistance. J. Appl. Pharm. Sci..

[9] Boparai J.K., Sharma P.K. (2020). Mini Review on Antimicrobial Peptides, Sources, Mechanism and Recent Applications. Protein Pept. Lett..

[10] Kang S.J., Park S.J., Mishig-Ochir T., Lee B.J. (2014). Antimicrobial peptides: Therapeutic potentials. Expert Rev. Anti. Infect. Ther..

[11] Haney E.F., Mansour S.C., Hancock R.E. (2017). Antimicrobial Peptides: An Introduction. Methods Mol. Biol..

[12] Lazzaro B.P., Zasloff M., Rolff J. (2020). Antimicrobial peptides: Application informed by evolution. Science.

[13] Lei J., Sun L., Huang S., Zhu C., Li P., He J., Mackey V., Coy D.H., He Q. (2019). The antimicrobial peptides and their potential clinical applications. Am. J. Transl. Res..

[14] Seo M.D., Won H.S., Kim J.H., Mishig-Ochir T., Lee B.J. (2012). Antimicrobial peptides for therapeutic applications: A review. Molecules.

[15] Boman H.G. (2003). Antibacterial peptides: Basic facts and emerging concepts. J. Intern. Med..

[16] Zasloff M. (2002). Antimicrobial peptides of multicellular organisms. Nature.

[17] Hancock R.E., Sahl H.G. (2006). Antimicrobial and host-defense peptides as new anti-infective therapeutic strategies. Nat. Biotechnol..

[18] Oyston P.C.F., Fox M.A., Richards S.J., Clark G.C. (2009). Novel peptide therapeutics for treatment of infections. J. Med. Microbiol..

[19] Otvos L. (2000). Antibacterial peptides isolated from insects. J. Pept. Sci..

[20] Reddy K.V., Yedery R.D., Aranha C. (2004). Antimicrobial peptides: Premises and promises. Int. J. Antimicrob. Agents.

[21] Falla T.J., Karunaratne D.N., Hancock R.E. (1996). Mode of action of the antimicrobial peptide indolicidin. J. Biol. Chem..

[22] Yan Y., Li Y., Zhang Z., Wang X., Niu Y., Zhang S., Xu W., Ren C. (2021). Advances of peptides for antibacterial applications. Colloids Surf. B. Biointerfaces.

[23] Tian T., Xie W., Liu L., Fan S., Zhang H., Qin Z., Yang C. (2021). Industrial application of antimicrobial peptides based on their biological activity and structure-activity relationship. Crit. Rev. Food Sci. Nutr..

[24] Laver D.R. (1994). The barrel-stave model as applied to alamethicin and its analogs reevaluated. Biophys. J..

[25] Buda De Cesare G., Cristy S.A., Garsin D.A., Lorenz M.C. (2020). Antimicrobial Peptides: A New Frontier in Antifungal Therapy. mBio.

[26] Cudic M., Otvos L. (2002). Intracellular targets of antibacterial peptides. Curr. Drug Targets.

[27] Luong H.X., Thanh T.T., Tran T.H. (2020). Antimicrobial peptides—Advances in development of therapeutic applications. Life Sci..

[28] Zhang Q.Y., Yan Z.B., Meng Y.M., Hong X.Y., Shao G., Ma J.J., Cheng X.R., Liu J., Kang J., Fu C.Y. (2021). Antimicrobial peptides: Mechanism of action, activity and clinical potential. Mil. Med. Res..

[29] Fjell C.D., Hiss J.A., Hancock R.E., Schneider G. (2011). Designing antimicrobial peptides: Form follows function. Nat. Rev. Drug Discov..

[30] Molhoek E.M., van Dijk A., Veldhuizen E.J., Haagsman H.P., Bikker F.J. (2011). Improved proteolytic stability of chicken cathelicidin-2 derived peptides by D-amino acid substitutions and cyclization. Peptides.

[31] Jia F., Wang J., Peng J., Zhao P., Kong Z., Wang K., Yan W., Wang R. (2017). D-amino acid substitution enhances the stability of antimicrobial peptide polybia-CP. Acta Biochim. Biophys. Sin..

[32] Li Y., Liu T., Liu Y., Tan Z., Ju Y., Yang Y., Dong W. (2019). Antimicrobial activity, membrane interaction and stability of the D-amino acid substituted analogs of antimicrobial peptide W3R6. J. Photochem. Photobiol. B..

[33] Zhao Y., Zhang M., Qiu S., Wang J., Peng J., Zhao P., Zhu R., Wang H., Li Y., Wang K. (2016). Antimicrobial activity and stability of the D-amino acid substituted derivatives of antimicrobial peptide polybia-MPI. AMB Express.

[34] Qiu S., Zhu R., Zhao Y., An X., Jia F., Peng J., Ma Z., Zhu Y., Wang J., Su J. (2017). Antimicrobial activity and stability of protonectin with D-amino acid substitutions. J. Pept. Sci..

[35] Chen H.L., Su P.Y., Shih C. (2016). Improvement of in vivo antimicrobial activity of HBcARD peptides by D-arginine replacement. Appl. Microbiol. Biotechnol..

[36] Lu J., Xu H., Xia J., Ma J., Xu J., Li Y., Feng J. (2020). D- and Unnatural Amino Acid Substituted Antimicrobial Peptides With Improved Proteolytic Resistance and Their Proteolytic Degradation Characteristics. Front. Microbiol..

[37] Alvares D.S., Wilke N., Ruggiero Neto J. (2018). Effect of N-terminal acetylation on lytic activity and lipid-packing perturbation induced in model membranes by a mastoparan-like peptide. Biochim. Biophys. Acta Biomembr..

[38] Dennison S.R., Mura M., Harris F., Morton L.H., Zvelindovsky A., Phoenix D.A. (2015). The role of C-terminal amidation in the membrane interactions of the anionic antimicrobial peptide, maximin H5. Biochim. Biophys. Acta.

[39] Sforça M.L., Oyama S., Canduri F., Lorenzi C.C., Pertinhez T.A., Konno K., Souza B.M., Palma M.S., Ruggiero N.J., Azevedo W.F. (2004). How C-terminal carboxyamidation alters the biological activity of peptides from the venom of the eumenine solitary wasp. Biochemistry.

[40] Zhang F., Guo Z.L., Chen Y., Li L., Yu H.N., Wang Y.P. (2019). Effects of C-terminal amidation and heptapeptide ring on the biological activities and advanced structure of amurin-9KY, a novel antimicrobial peptide identified from the brown frog, Rana kunyuensis. Zool. Res..

[41] Li D., Yang Y., Li R., Huang L., Wang Z., Deng Q., Dong S. (2021). N-terminal acetylation of antimicrobial peptide L163 improves its stability against protease degradation. J. Pept. Sci..

[42] Zhu S., Li W., O’Brien-Simpson N., Separovic F., Sani M.A. (2021). C-terminus amidation influences biological activity and membrane interaction of maculatin 1.1. Amino Acids.

[43] Vineeth Kumar T., Asha R., George S. (2021). Identification and functional characterisation of Esculentin-2 HYba peptides and their C-terminally amidated analogs from the skin secretion of an endemic frog. Nat. Prod. Res..

[44] Kuzmin D.V., Emelianova A.A., Kalashnikova M.B., Panteleev P.V., Ovchinnikova T.V. (2017). Effect of N- and C-Terminal Modifications on Cytotoxic Properties of Antimicrobial Peptide Tachyplesin I. Bull. Exp. Biol. Med..

[45] Moiola M., Memeo M.G., Quadrelli P. (2019). Stapled Peptides-A Useful Improvement for Peptide-Based Drugs. Molecules.

[46] Migoń D., Neubauer D., Kamysz W. (2018). Hydrocarbon Stapled Antimicrobial Peptides. Protein J..

[47] Verdine G.L., Hilinski G.J. (2012). Stapled peptides for intracellular drug targets. Methods Enzymol..

[48] Hirano M., Saito C., Yokoo H., Goto C., Kawano R., Misawa T., Demizu Y. (2021). Development of Antimicrobial Stapled Peptides Based on Magainin 2 Sequence. Molecules.

[49] Liu J., Chen S., Chai X.Y., Gao F., Wang C., Tang H., Li X., Liu Y., Hu H.G. (2021). Design, synthesis, and biological evaluation of stapled ascaphin-8 peptides. Bioorg. Med. Chem..

[50] Scala M.C., Spensiero A., Pepe G., Bertamino A., Carotenuto A., Grieco P., Novellino E., Gomez-Monterrey I.M., Campiglia P., Sala M. (2018). Investigation on side-product formation during the synthesis of a lactoferrin-derived lactam-bridged cyclic peptide. Amino Acids.

[51] Zhang R.Y., Thapa P., Espiritu M.J., Menon V., Bingham J.P. (2018). From nature to creation: Going around in circles, the art of peptide cyclization. Bioorg. Med. Chem..

[52] Neubauer D., Jaśkiewicz M., Sikorska E., Bauer S.B.M., Kapusta M., Narajczyk M., Kamysz W. (2020). Effect of Disulfide Cyclization of Ultrashort Cationic Lipopeptides on Antimicrobial Activity and Cytotoxicity. Int. J. Mol. Sci..

[53] Browne K., Chakraborty S., Chen R., Wilcox M.D.P., Black D.S., Walsh W.R., Kumer N. (2020). A New Era of Antiboitics: The Clinical Potential of Antimicrobial Peptides. Int. J. Mol. Sci..

[54] Kang S.J., Park S.J., Lee B.J. (2009). De novo generation of antimicrobial LK peptides with a single trypophan at the critical anphipathic interface. J. Pept. Sci..

[55] Wang C., Yang C., Chen Y.C., Ma L., Huang K. (2019). Rational Design of Hybrid Peptides: A Novel Drug Design Approach. Curr. Med. Sci..

[56] Khardori N., Stevaux C., Ripley K. (2020). Antibiotics: From the Beginning to the Future: Part 2. Indian J. Pediatr..

[57] Almaaytah A., Qaoud M.T., Abualhaijaa A., Al-Balas Q., Alzoubi K.H. (2018). Hybridization and antibiotic synergism as a tool for reducing the cytotoxicity of antimicrobial peptides. Infect. Drug Resist..

[58] Fox M.A., Thwaite J.E., Ulaeto D.O., Atkins T.P., Atkins H.S. (2012). Design and characterization of novel hybrid antimicrobial peptides based on cecropin A, LL-37 and magainin II. Peptides.

[59] David A.A., Park S.E., Parang K., Tiwari R.K. (2018). Antibiotics-Peptide Conjugates Against Multidrug-resistant Bacterial Pathogens. Curr. Top Med. Chem..

[60] Chen H., Liu C., Chen D., Madrid K., Peng S., Dong X., Zhang M., Gu Y. (2015). Bacteria-Targeting Conjugates Based on Antimicrobial Peptide for Bacteria Diagnosis and Therapy. Mol. Pharm..

[61] Arnusch C.J., Pieters R.J., Breukink E. (2012). Enhanced membrane pore formation through high-affinity targeted antimicrobial peptides. PLoS ONE.

[62] Etayash H., Alford M., Akhoundsadegh N., Drayton M., Straus S.K., Hancock R.E.W. (2021). Multifunctional Antibiotic-Host Defense Peptide Conjugate Kills Bacteria, Eradicates Biofilms, and Modulates the Innate Immune Response. J. Med. Chem..

[63] Wang Z., Liu X., Da T., Mao R., Hao Y., Yang N., Wang X., Li Z., Wang X., Wang J. (2020). Development of chimeric peptides to facilitate the neutralisation of lipopolysaccharides during bactericidal targeting of multidrug-resistant Escherichia coli. Commun. Biol..

[64] Lee H., Lim S.I., Shin S.H., Lim Y., Koh J., Yang S. (2019). Conjugation of Cell-Penetrating Peptides to Antimicrobial Peptides Enhances Antibacterial Activity. ACS Omega.

[65] Li Z., Wang X., Teng D., Mao R., Hao Y., Yang N., Chen H., Wnag X., Wnag J. (2018). Improved antibacterial activity of a marine peptide-N2 against intracellular *Samonella typhimurium* by conjugating with cell-penetrating peptides-bLFcin_6_/Tat_11_. Eur. J. Med. Chem..

[66] Cardoso P., Glossop H., Meikle T.G., Aburto-Medina A., Conn C.E., Sarojini V., Valery C. (2021). Molecular engineering of antimicrobial peptides: Microbial targets, peptide motifs and translation poortunities. Biophys. Rev..

[67] Reonhardt A., Neundorf I. (2016). Design and Application of Antimicrobial Peptide Conjugates. Int. J. Mol. Sci..

[68] Biswaro L.S., da Costa Sousa M.G., Rezende T.M.B., Dias S.C., Franco O.L. (2018). Antimicrobial Peptides and Nanotechnology, Recent Advances and Challenges. Front. Microbiol..

[69] Tang Z., Ma Q., Chen X., Chen T., Ying Y., Xi X., Wang L., Ma C., Shaw C., Zhou M. (2021). Recent Advances and Challenges in Nanodelivery Systems for Antimicrobial Peptides (AMPs). Antibiotics.

[70] Rajchakit U., Sarojini V. (2017). Recent developments in antimicrobial-peptide-conjugated gold nanoparticles. Bioconjugate Chem..

[71] Bruna T., Maldonado-Bravo F., Jara P., Caro N. (2021). Silver Nanoparticles and Their Antibacterial Applications. Int. J. Mol. Sci..

[72] Kukushkina E.A., Hossain S.I., Sportelli M.C., Ditaranto N., Picca R.A., Cioffi N. (2021). Ag-Based Synergistic Antimicrobial Composites. A Critical Review. Nanomaterials.

[73] Zharkova M.S., Golubeva O.Y., Orlov D.S., Vladimirova E.V., Dmitriev A.V., Tossi A., Shamova O.V. (2021). Silver Nanoparticles Functionalized With Antimicrobial Polypeptides: Benefits and Possible Pitfalls of a Novel Anti-infective Tool. Front. Microbiol..

[74] Pal I., Bhattacharyya D., Kar R.K., Zarena D., Bhunia A., Atreya H.S. (2019). A Peptide-Nanoparticle System with Improved Efficacy against Multidrug Resistant Bacteria. Sci. Rep..

[75] Lee B., Park J., Ryu M., Kim S., Joo M., Yeom J.H., Kim S., Park Y., Lee K., Bae J. (2017). Antimicrobial peptide-loaded gold nanoparticle-DNA aptamer conjugates as highly effective antibacterial therapeutics against Vibrio vulnificus. Sci. Rep..

[76] Casciaro B., Moros M., Rivera-Fernández S., Bellelli A., de la Fuente J.M., Mangoni M.L. (2017). Gold-nanoparticles coated with the antimicrobial peptide esculentin-1a(1-21)NH(2) as a reliable strategy for antipseudomonal drugs. Acta Biomater..

[77] Olusanya T.O.B., Haj Ahmad R.R., Ibegbu D.M., Smith J.R., Elkordy A.A. (2018). Liposomal Drug Delivery Systems and Anticancer Drugs. Molecules.

[78] Cantor S., Vargas L., Rojas A.O.E., Yarce C.J., Salamanca C.H., Oñate-Garzón J. (2019). Evaluation of the Antimicrobial Activity of Cationic Peptides Loaded in Surface-Modified Nanoliposomes against Foodborne Bacteria. Int. J. Mol. Sci..

[79] Ron-Doitch S., Sawodny B., Kühbacher A., David M.M.N., Samanta A., Phopase J., Burger-Kentischer A., Griffith M., Golomb G., Rupp S. (2016). Reduced cytotoxicity and enhanced bioactivity of cationic antimicrobial peptides liposomes in cell cultures and 3D epidermis model against HSV. J. Control Release.

[80] Rai A., Rerrao R., Palma P., Patrocop T., Parreira P., Anes E., Tonda-Turo C., Martins M.C.L., Alves N., Ferreira L. (2022). Antimicrobial peptide-based materials: Opportunities and challenges. J. Mater. Chem. B.

[81] Cleophas R.T.C., Riool M., Quarles van Ufford H.C., Zaat S.A.J., Kruijtzer J.A.W., Liskamp M.J. (2014). Convenient Preparation of Bactericidal Hydrogels by Covalent Attachment of Stabilized Antimicrobial Peptides Using Thio-ene Click Chemistry. ACS Macro Lett..

[82] Almaaytah A., Mohammed G.K., Abualhaijaa A., Al-Balas Q. (2017). Development of novel ultrashort antimicrobial peptide nanoparticles with potent antimicrobial and antibiofilim activities against multidrug-resistant bactera. Drug Des. Devel. Ther..

[83] Yu C.Y., Huang W., Li Z.P., Lei X.Y., He D.X., Sun L. (2016). Progress in Self-assembling Peptide-based Nanomaterials for Biomedical Applications. Curr. Top Med. Chem..

[84] Innocenti Malini R., Zabara M., Gontsarik M., Maniura-Weber K., Rossi R.M., Spano F., Salentinig S. (2020). Self-assembly of glycerol monooleate with the antimicrobial peptide LL-37: A molecular dynamics study. RSC Adv..

[85] Lei R., Hou J., Chen Q., Yuan W., Cheng B., Sun Y., Jin Y., Ge L., Ben-Sasson S.A., Chen J. (2018). Self-Assembling Myristoylated Human α-Defensin 5 as a Next-Generation Nanobiotics Potentiates Therapeutic Efficacy in Bacterial Infection. ACS Nano.

[86] Yang Z., He S., Wu H., Yin T., Wang L., Shan A. (2021). Nanostructured Antimicrobial Peptides: Crucial Steps of Overcoming the Bottleneck for Clinics. Front. Microbiol..

